# Supplementary material for: Host/Malassezia Interaction: A Quantitative, Non-Invasive Method Profiling Oxylipin Production Associates Human Skin Eicosanoids with Malassezia
Source: Metabolites. 2021 Oct 13;11(10):700. doi: 10.3390/metabo11100700 (PMC8538739; doi:10.3390/metabo11100700)
Supplement: Supplementary file 1 [file metabolites-11-00700-s001.zip › metabolites-1375203-supplementary/Supplementary Material_10102021.pdf]

Supporting Materials

Host/*Malassezia* Interaction: A Quantitative, Non-Invasive Method Profiling Oxylipin Production Associates Human Skin Eicosanoids with *Malassezia*

Yohannes Abere Ambaw [1,2,3†], Martin P. Pagac [4,†], Antony S. Irudayaswamy [4], Manfred Raida [2], Anne K. Bendt [2],
Federico Torta [1,2], Markus R. Wenk [1,2] and Thomas L. Dawson, Jr. [4,5,*]

Section S1:

1. Lipid mediator extraction from flax-paper

Oxylipins extraction was carried out according to the previous publication with small modifications. Briefly, lipids were released from flax paper disks overnight at 4°C with 1 mL of methanol (MeOH): water (H_2O) (5:95 by v/v) at 900 rpm (revolution per minute) in a thermomixer (Eppendorf, Hamburg, Germany). Samples were spiked with 50 µl of an optimized, mixed deuterated internal standard solution (see Table S2). Oxylipins extraction was performed with some modifications of an earlier method [1]. Analytes were extracted using Strata-X 33 u polymer based solid reverse phase (SPE) extraction columns (8B-S100-UBJ, Phenomenex). Columns were conditioned with 3 mL of 100% MeOH and then equilibrated with 3 mL of H_2O. After loading the sample, the columns were washed with H_2O: MeOH: acetic acid (90:10:0.1 by v/v) to remove impurities, and the metabolites were then eluted with 2 times 500 µl of 100 % MeOH. The eluant was dried under vacuum and redissolved in 50 µl of the ACN/water/acetic acid (60/40/0.02, v/v). The extracted samples were then subjected to mass spectrometry analysis.

2. Analysis of lipid mediators from cultured *Malassezia*

All *Malassezia* strains were cultured in modified Dixon media (per liter water: 36 g malt extract, 20 g desiccated ox bile, 10 ml Tween-40s, 6 g peptone, 2 ml glycerol, 2 ml oleic acid, pH 6.0) as

reported previously [2]. *Malassezia* strains reported here are *M. globosa* CBS 7996, *M. furfur* CBS14141, *M. sympodialis* CBS 7222. 15 ml of triplicate cultures were grown in a shaker incubator at 32 °C to late exponential growth phase and harvested by centrifugation, washed three times in PBS, transferred to 2 ml Eppendorf tubes and stored at -80°C prior to lipid extraction. The resulting ~1 ml cell pellets were lyophilized in a speedvac and weighted. For lipid extraction, the cell pellet was resuspended in 1 ml water (pH 4.48), acid-washed glass beads were added and the suspension was homogenized by shaking overnight at 900 rpm in a thermomixer at 4°C. The following day, cells were further homogenized in a TissueLyser using standard parameters (2 times for 30 seconds at 5.15 meters/second speed, 4 °C). After a quick centrifugation, the supernatants were transferred into new 2 ml Eppendorf tubes, 100 µl Methanol and ISTD mix were added, followed by a quick centrifugation step. Supernatants were transferred into new 2 ml Eppendorf tubes, 1 ml of cold water (pH 4.48) was added, and lipids were extracted by SPE as described above. Lipidomic analysis was performed by LC-MS/MS using Agilent 6490 mass spectrometer as described above, and lipid concentrations were normalized to previously determined cell pellet dry weights.

Section S2:

Targeted LC-MS/MS analysis of lipid mediators

Lipidomic analysis was performed using LC electrospray ionisation MS/MS (LC-ESI-MS/MS) as previously described [3][4][5][1, 2]. Briefly, an Agilent 6495 triple quadrupole (QQQ) mass spectrometer (Agilent 1290 series HPLC system and a Acquity UPLC BEH shield RP18 column (2.1× 100 mm; 1.7 m; Waters) in negative ion mode was used. The solvent system consisted of solvent A, ACN/water/acetic acid (60/40/0.02, v/v), and solvent B, 1 ACN/IPA (50/50, v/v). The stepwise gradient conditions were carried out for 10 min with flow rate was 0.5 mL/min as follows:

0–5.0 min, 1–55% of solvent B; 5.0–5.5 min, 55–99% of solvent B, and finally 5.5–6.0 min, 99% of solvent B. We used an injection volume was 10 µl, and all samples were kept at 4 ˚C throughout the analysis. The following mass spectrometer conditions were used: gas temperature, 250˚C; gas flow rate, 14 L/min; nebuliser, 35 psi; sheath gas temperature, 260˚C; capillary voltage, 10 V; and sheath gas flow, 14 L/min. The dynamic MRM option was used and performed for all compounds with optimized transitions and collision energies (see Table S1). The determination and integration of all peaks was manually performed using the MassHunter Workstation software (Agilent, Santa Clara, USA). Peaks were smoothed before integration and peak to peak Signal/Noise ratios were determined using the area under the peaks.

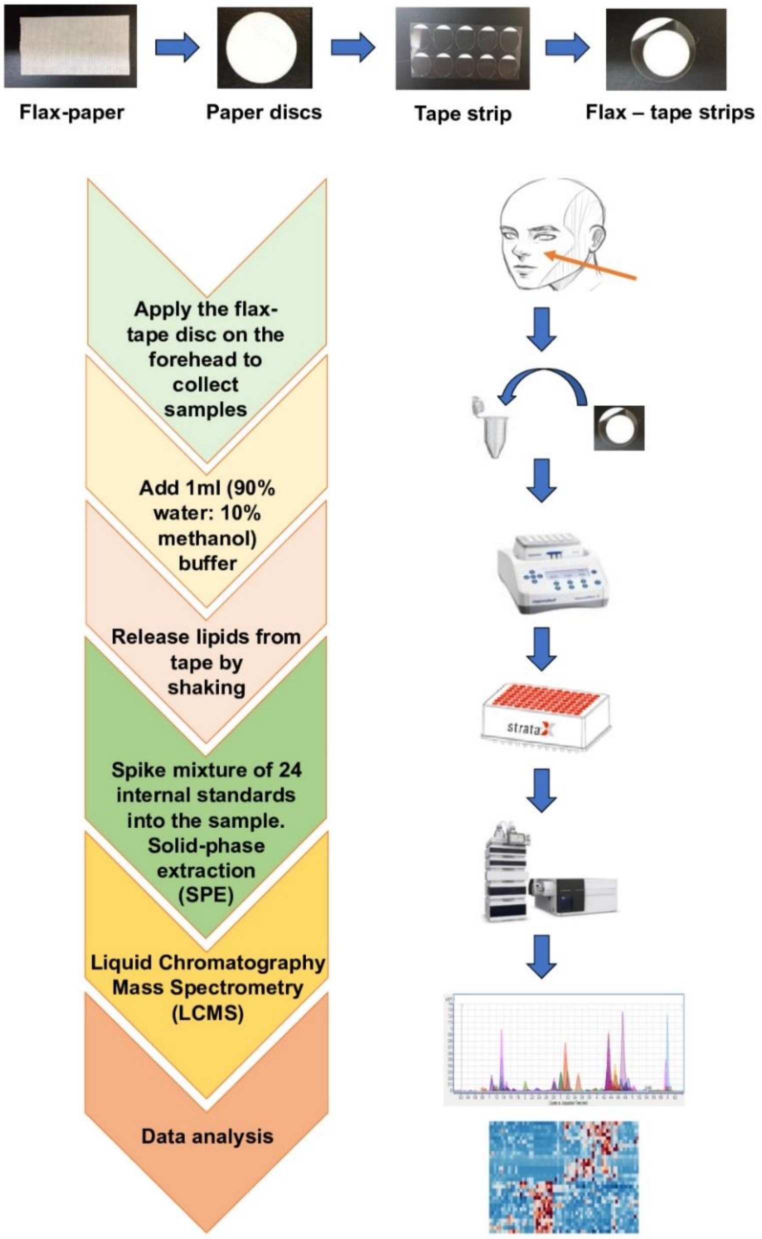

Figure S1: Workflow for lipid mediator analysis. The flow chart showing the main steps sample collection, extraction, instrumental analysis and data analysis.

S2

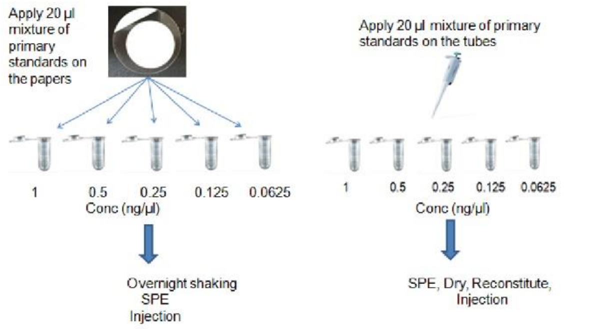

S3

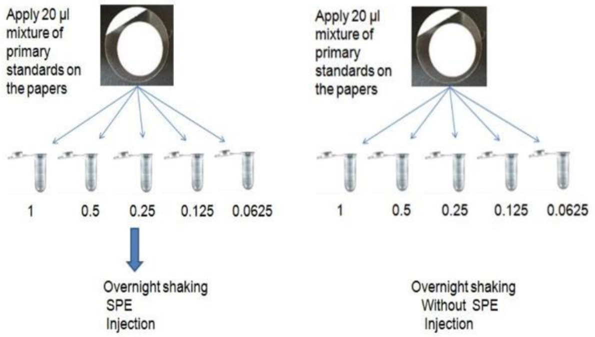

Figure S2. Schematic diagram illustrating the procedures for validating the lipid absorbance level of flax-disk-paper. Briefly, different concentrations of mixed non-deuterated standards were applied onto the flax-disk paper and lipids were then extracted. Experiments were performed in triplicates and analyzed.

Figure S3. Schematic diagram illustrating the procedures for validating the recovery rate of flax-disk paper and SPE (solid phase extraction). Briefly, different concentrations of mixed non-deuterated standards were applied onto the flax-disk paper and lipids were then extracted. Experiments were performed in triplicates and analyzed.

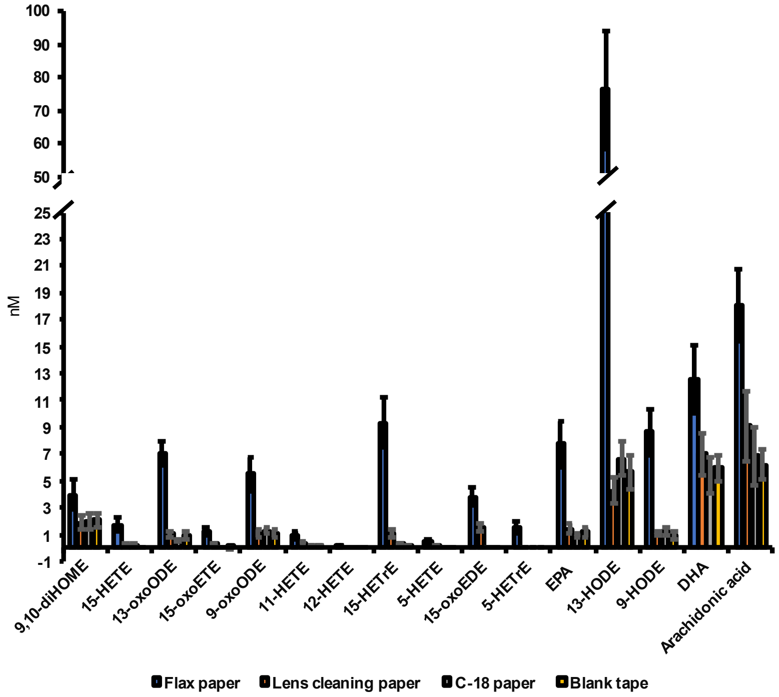

Figure S4. Selection of the optimal lipid mediator absorbent. Absorption difference between a subset of trial materials. Flax paper absorbs significantly higher lipid content from human skin (n=3, data indicates in mean ± SD).

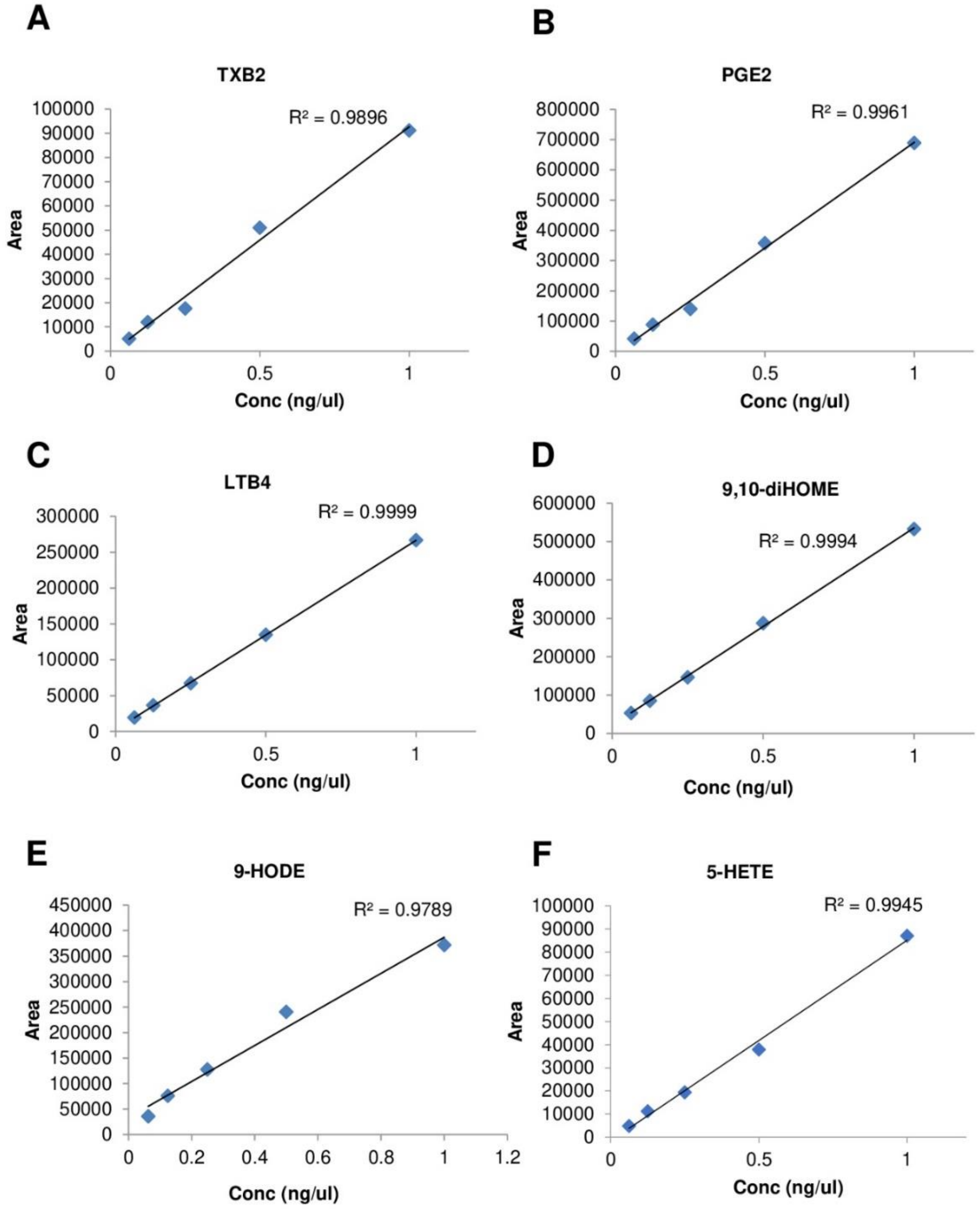

Figure S5. Linearity of representative standards TXB2, PGE2, LXB_4, 9,10-diHOME, 9-HODE and 5-HETE respectively. Y axis indicate ratio of analyte peak area and X axis indicate the concentration of the analyte standard. All lipid mediators were detected and successfully quantified with satisfactory linearity with R^2 value greater than 0.98, suggesting that flax paper is able to quantitatively absorb skin lipid mediators in a relevant linear range without saturation.

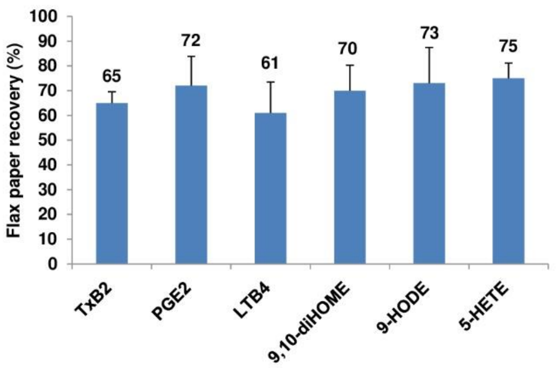

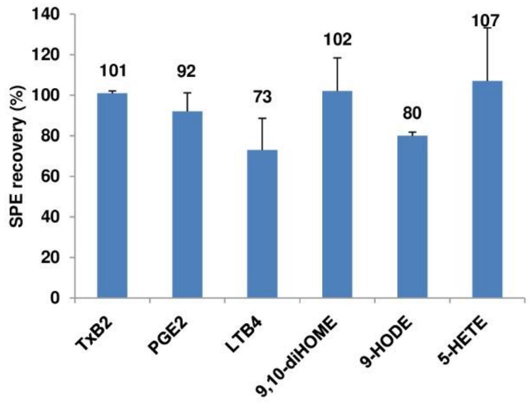

Figure S6. Recovery rate of representative standards TXB2, PGE2, LXB_4, 9,10-diHOME, 9-HODE and 5-HETE respectively. Y axis indicate % recovery rate of indicated standards from flax paper followed by SPE.

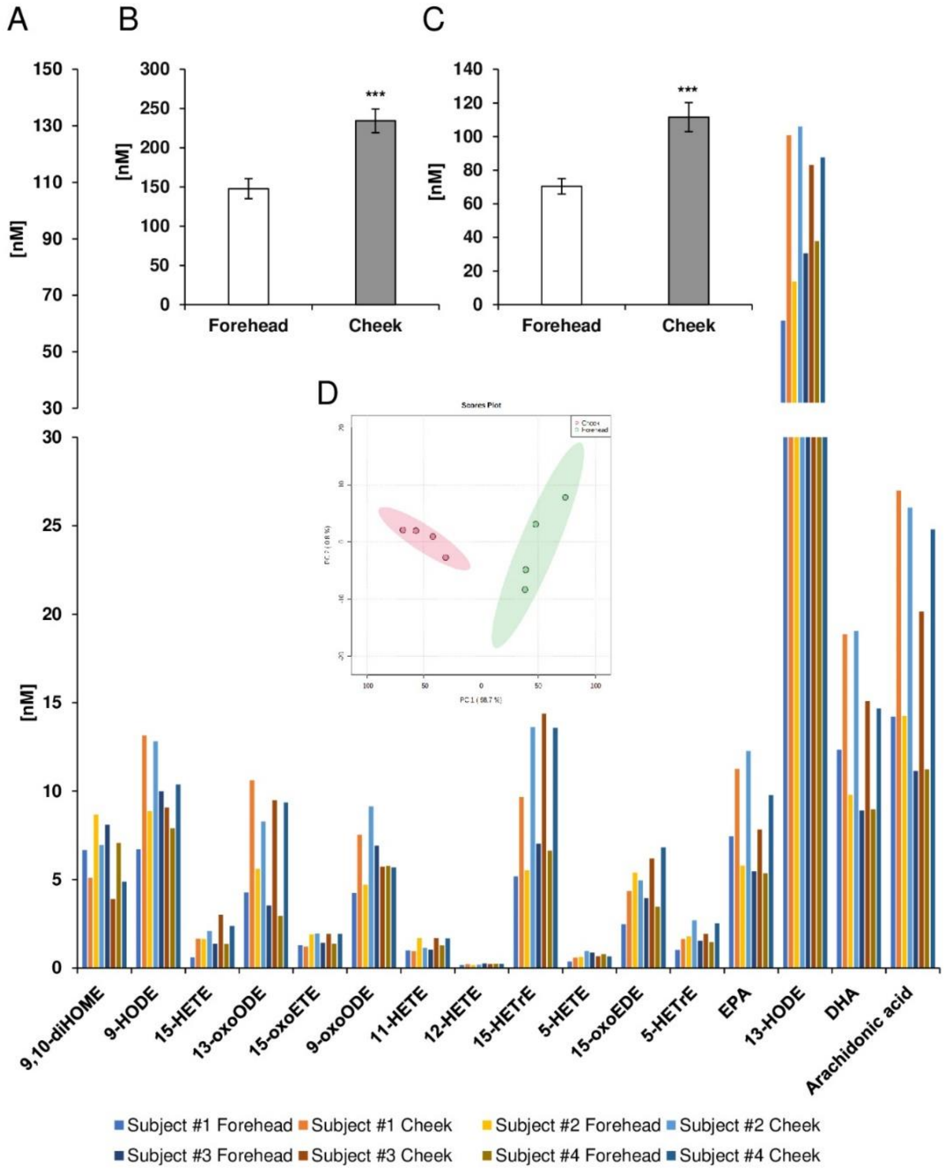

Figure S7: A) Direct comparison of facial skin lipid mediator concentrations captured on cheek and forehead by using flax paper disks (n=4). B) Total concentrations of lipid mediators detected on cheek are significantly higher than on forehead. C) Same as B), except that the most abundant oxylipin species 13-HODE was excluded (*** $p \leq 0.001$). D) Principal component analysis (PCA) comparing lipid mediator concentrations between cheek and forehead reveals a strong intra-individual variability.

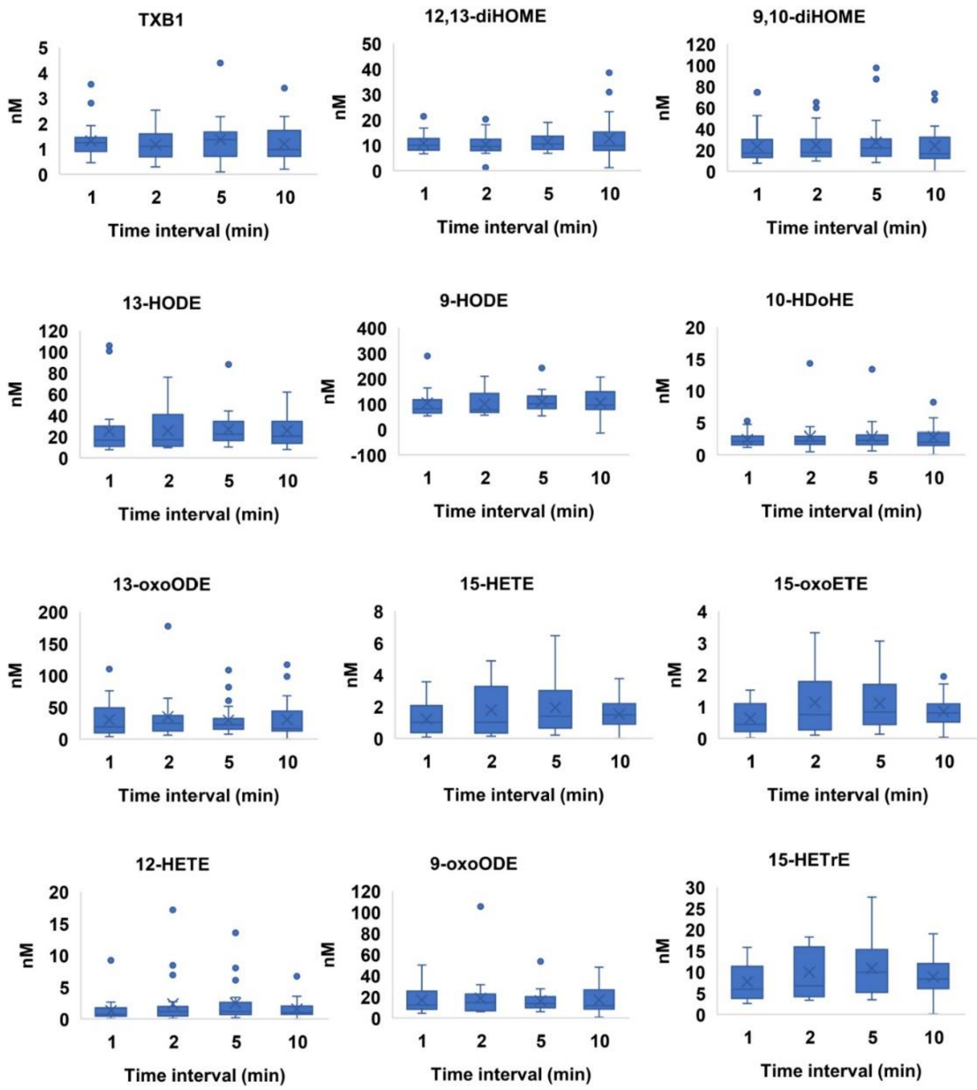

Figure S8. Lipid mediator capture difference with time interval between 1-, 2-, 5- and 10-mins collection. Scatter plots showing the median (bold horizontal line), interquartile rang (box) and oxylipin concertation levels in the Y-axis across four different pre-selected time intervals (0, 1, 5 and 10 mins). Results are the mean ± S.E.

Table S1. List of MRM transitions for eicosanoids species, and non-natural compounds used as internal standards (ISTD). An appropriate internal standard for the quantification of each endogenous lipid is listed. The retention time and collision energy is also reported for each compound. (M1 = precursor ion; M2 = product ion; RT=Retention time in minute and CE=collision energy).

**Compound Name**	**M1**	**M2**	**ISTD**	**RT**	**CE**
(d4) 6k PGF1	373	167	ISTD	0.87	25
(d4) TXB_2	373	173	ISTD	1.17	13
(d4) $PGF_{2\alpha}$	357	197	ISTD	1.26	25
(d4) PGE_2	355	275	ISTD	1.31	13
(d4) PGD_2	355	193	ISTD	1.47	13
(d4) dhk $PGF_{2\alpha}$	357	187	ISTD	1.68	21
(d4) dhk PGD_2	355	179	ISTD	1.94	19
(d4) 5-iso $PGF_{2\alpha}$ VI	364	115	ISTD	1.14	21
(d4) LTB_4	339	197	ISTD	2.79	13
(d8) 5-HETE	327	116	ISTD	4.65	13
(d8) 12-HETE	327	184	ISTD	4.47	13
(d8) 15-HETE	327	226	ISTD	4.22	13
(d4) 9-HODE	299	172	ISTD	4.29	19
(d4) 13-HODE	299	198	ISTD	4.28	13
(d4) Resolvin E1	353	197	ISTD	0.96	13
(d11) 8,9-EET	330	155	ISTD	4.92	9
(d11) 11,12-DHET	348	167	ISTD	3.4	19
(d11) 14,15-EET	330	175	ISTD	4.78	9
(d4) 9,10-diHOME	317	203	ISTD	3.15	21
(d4) 12,13-diHOME	317	185	ISTD	3.05	21
(d5) LTE4	443	338	ISTD	2.1	17
(d8) Arachidonic acid	311	267	ISTD	5.97	13
TxB_2	369	169	(d4) TXB_2	1.12	13
$PGF_{2\alpha}$	353.3	193	(d4) $PGF_{2\alpha}$	1.2	25
PGE_2	351	271	(d4) PGE_2	1.31	13
PGD_2	351	271	(d4) PGD_2	1.49	13
tetranor 12-HETE	265	109	(d4) LTB_4	3.11	9
12-HHTrE	279	217	(d4) LTB_4	3.33	13
11-HETE	319	167	(d8) 5-HETE	4.43	13
11-HEPE	317	167	(d8) 5-HETE	3.79	13
13-HDoHE	343	221	(d8) 15-HETE	4.41	13
9-HETE	319	123	(d8) 5-HETE	4.54	13
9-HEPE	317	149	(d8) 5-HETE	4.1	13
8-HDoHE	343	109	(d8) 5-HETE	4.55	13
16-HDoHE	343	233	(d8) 15-HETE	4.34	13
20-HDoHE	343	241	(d8) 15-HETE	4.24	9

LTB_4	335	195	(d4) LTB_4	2.79	13
6-trans-LTB_4	335	195	(d4) LTB_4	2.52	13
5,6-diHETE	335	115	(d4) LTB_4	3.84	21
5-HETE	319	115	(d8) 5-HETE	4.66	13
5-HEPE	317	115	(d8) 5-HETE	4.05	13
7-HDoHE	343	141	(d8) 5-HETE	4.54	13
4-HDoHE	343	101	(d8) 5-HETE	4.81	9
9-HOTrE	293	171	(d8) 5-HETE	3.66	13
6S-LXA_4	351.3	115	(d4) LTB_4	1.89	9
Resolvin E1	349	195	(d4) Resolvin E1	0.89	13
Resolvin D1	375	141	(d4) Resolvin E1	1.74	13
Protectin D1	359	153	(d4) Resolvin E1	1.85	13
8,15-diHETE	335	235	(d4) LTB_4	2.51	19
15-HETE	319	175	(d8) 15-HETE	4.34	13
15-HEPE	317	219	(d8) 5-HETE	3.81	13
17 HDoHE	343	229	(d8) 15-HETE	4.32	9
13-HODE	295	195	(d4) 13-HODE	4.27	13
13-HOTrE	293	195	(d4) 13-HODE	3.85	19
15-HETrE	321	221	(d8) 15-HETE	4.69	13
8-HETE	319	155	(d8) 5-HETE	4.54	9
8-HEPE	317	155	(d8) 5-HETE	4.04	21
10-HDoHE	343	153	(d8) 5-HETE	4.45	9
8-HETrE	321	157	(d8) 5-HETE	4.72	13
12-HETE	319	135	(d8) 12-HETE	4.43	13
12-HEPE	317	179	(d8) 12-HETE	3.88	9
14-HDoHE	343	205	(d8) 15-HETE	4.43	9
11-HDoHE	343	149	(d8) 15-HETE	4.58	11
9-HODE	295	171	(d4) 9-HODE	4.26	19
12-oxoETE	317	153	(d8) 15-HETE	4.42	13
15-oxoETE	317	113	(d8) 15-HETE	4.33	13
20-HETE	319.3	275	(d8) 15-HETE	3.84	13
19-HETE	319	231	(d8) 15-HETE	3.78	13
18-HETE	319	261	(d8) 15-HETE	3.82	9
17-HETE	319	247	(d8) 15-HETE	4.11	9
16-HETE	319	189	(d8) 15-HETE	4.03	9
18-HEPE	317	215	(d8) 15-HETE	3.7	9
5,6-EET	319	191	(d11) 8,9-EET	5.19	9
8,9-EET	319	123	(d11) 8,9-EET	5.07	9
11,12-EET	319	167	(d11) 14,15-EET	4.95	9
14,15-EET	319	219	(d11) 14,15-EET	4.78	9
19(20)-EpDPE	343	241	(d8) 15-HETE	4.7	9
19,20-DiHDPA	361	229	(d11) 11,12-DHET	3.28	13
5,6-diHETrE	337.3	145	(d11) 11,12-DHET	3.82	13

8,9-diHETrE	337.2	127	(d11) 11,12-DHET	3.65	19
11,12-diHETrE	337.3	167	(d11) 11,12-DHET	3.53	13
14,15-diHETrE	337.3	207	(d11) 11,12-DHET	3.24	13
9,10-diHOME	313	201	(d4) 9,10-diHOME	3.19	21
12,13-diHOME	313	183	(d4) 12,13-diHOME	3	21
9,10-EpOME	295	171	(d11) 8,9-EET	4.8	13
12,13-EpOME	295	195	(d11) 8,9-EET	4.75	13
Arachidonic acid	303	259	(d8) Arachidonic acid	5.92	13
Adrenic acid	331	287	(d8) Arachidonic acid	6.03	13
EPA	301	257	(d8) Arachidonic acid	5.78	9
DHA	327	283	(d8) Arachidonic acid	5.93	9

Table S2. Oxylipins internal standards and their concentrations in MS analysis

**Internal standards**	**Conc. (nM)**
(d4) 6k PGF1	6
(d4) dhk PGF2	6
(d4) PGF2	6
(d5) LTC4	3.5
(d5) LTE4	5
(d4) Resolvin E1	6
(d4) PGD2	6
(d4) 5-iso PGF2 VI	6
(d8) 5-HETE	7
(d6) 20-HETE	7
(d7) 5-oxoETE	7
(d8) 12-HETE	7
(d8) 15-HETE	7
(d4) 12,13-diHOME	2
(d11) 11,12-DHET	1.5
(d4) PGE2	1
(d4) LTB4	3
(d4) 13-HODE	1.5
(d4) 9-HODE	1.5
(d4) 9,10-diHOME	1.5
(d4) TXB2	2
(d4) dhk PGD2	4.5
(d11) 8,9-EET	7
(d11) 14,15-EET	7

(d8) Arachidonic acid	10

References

1. Y. Wang, A. M. Armando, O. Quehenberger, C. Yan, and E. A. Dennis, “Comprehensive ultra-performance liquid chromatographic separation and mass spectrometric analysis of eicosanoid metabolites in human samples,” *J. Chromatogr. A*, vol. 1359, pp. 60–69, Sep. 2014.
2. Y. M. DeAngelis *et al.*, “Isolation and Expression of a Malassezia globosa Lipase Gene, LIP1,” *J. Invest. Dermatol.*, vol. 127, no. 9, pp. 2138–2146, Sep. 2007.
3. Y. A. Ambaw *et al.*, “Tear eicosanoids in healthy people and ocular surface disease,” *Sci. Rep.*, 2018.
4. Y. A. Ambaw *et al.*, “Changes of tear lipid mediators after eyelid warming or thermopulsation treatment for meibomian gland dysfunction.,” *Prostaglandins Other Lipid Mediat.*, vol. 151, p. 106474, Dec. 2020.
5. Y. A. Ambaw, D. P. Timbadia, M. Raida, F. Torta, M. R. Wenk, and L. Tong, “Profile of tear lipid mediator as a biomarker of inflammation for meibomian gland dysfunction and ocular surface diseases: Standard operating procedures,” *Ocul. Surf.*, Oct. 2020.
6. Ambaw, Y.A., et al., Change of tear lipid mediators in a post-trabeculectomy cohort. Ocul Surf, 2020. **18**(4): p. 565-574
7. Ambaw, Y.A., et al., Tailored Polymer-Based Selective Extraction of Lipid Mediators from Biological Samples. Metabolites, 2021. **11**(8): p. 539.
